# Gaps in U.S. livestock data are a barrier to effective environmental and disease management

**DOI:** 10.1088/1748-9326/adb050

**Published:** 2025-02-11

**Authors:** Rebecca Logsdon Muenich, Sanskriti Aryal, Amanda J Ashworth, Michelle L Bell, Melanie R Boudreau, Stephanie A Cunningham, K Colton Flynn, Kerry A Hamilton, Ting Liu, Michael L Mashtare, Natalie G Nelson, Barira Rashid, Arghajeet Saha, Danica Schaffer-Smith, Callie Showalter, Aureliane Tchamdja, Jada Thompson

**Affiliations:** 1 Biological and Agricultural Engineering, University of Arkansas, Fayetteville, AR, United States of America; 2 Science and Technologies for Phosphorus Sustainability, Raleigh, NC, United States of America; 3 Quantitative Ecology and Spatial Technologies Laboratory, Department of Wildlife, Fisheries, and Aquaculture, Mississippi State University, Mississippi State, MS, United States of America; 4 United States Department of Agriculture, Agricultural Research Service, Poultry Production and Product Safety Research Unit, Fayetteville, AR, United States of America; 5 School of the Environment, Yale University, New Haven, CT, United States of America; 6 School of Health Policy and Management, College of Health Sciences, Korea University, Seoul 02841, Republic of Korea; 7 United States Department of Agriculture, Grassland, Soil and Water Research Laboratory, Temple, TX, United States of America; 8 School of Sustainable Engineering & the Built Environment, Arizona State University, Tempe, AZ, United States of America; 9 Department of Agricultural and Biological Engineering, The Pennsylvania State University, University Park, PA, United States of America; 10 Biological and Agricultural Engineering, North Carolina State University, Raleigh, NC, United States of America; 11 Center for Geospatial Analytics, North Carolina State University, Raleigh, NC, United States of America; 12 Kansas Geological Survey, University of Kansas, Lawrence, KS, United States of America; 13 School of Life Sciences, Arizona State University, Tempe, AZ, United States of America; 14 Department of Environmental Science and Policy, Smith College, Northampton, MA, United States of America; 15 Department of Computer Science, University of Maryland Baltimore County, Baltimore, MD, United States of America; 16 Agricultural Economics and Agribusiness, University of Arkansas, Fayetteville, AR, United States of America

**Keywords:** livestock, pollution, climate change, nutrient management, zoonotic disease risk, manure

## Abstract

Livestock are a critical part of our food systems, yet their abundance globally has been cited as a driver of many environmental and human health concerns. Issues such as soil, water, and air pollution, greenhouse gas emissions, aquifer depletion, antimicrobial resistance genes, and zoonotic disease outbreaks have all been linked to livestock operations. While many studies have examined these issues at depth at local scales, it has been difficult to complete studies at regional or national scales due to the dearth of livestock data, hindering pollution mitigation or response time for tracing and monitoring disease outbreaks. In the U.S. the National Agricultural Statistics Service completes a Census once every 5 years that includes livestock, but data are only available at the county level leaving little inference that can be made at such a coarse spatiotemporal scale. While other data exist through some regulated permitting programs, there are significant data gaps in where livestock are raised, how many livestock are on site at a given time, and how these livestock and, importantly, their waste emissions, are managed. In this perspective, we highlight the need for better livestock data, then discuss the accessibility and key limitations of currently available data. We then feature some recent work to improve livestock data availability through remote-sensing and machine learning, ending with our takeaways to address these data needs for the future of environmental and public health management.

## 1. The importance of livestock data

There are more than three-times the number of livestock on Earth as there are humans (Robinson *et al* 2014), and they are a vital part of our food and fiber system. As the global population has risen, so has demand for livestock and livestock-derived products. The subsequent rise in livestock numbers to meet this demand, along with economic drivers and cultural changes, has resulted in a move away from free-grazing systems to confined animal facilities, also known as concentrated animal feeding operations (CAFOs), intensive livestock facilities, feedlots, etc, where the animals are given a primarily grain-fed diet rather than free-grazing (MacDonald *et al* 2018). While many studies evaluate the trade-offs between confined and grazing systems, few studies have focused on the need for a better accounting of where, when, and how livestock are raised.

Despite being integral to our agroecosystems, livestock can have undesirable impacts on environmental and human health. Livestock can be sources of water and soil pollution through the emission of wastes which can contain excess nutrients, pathogens, antimicrobial resistance genes, veterinary pharmaceuticals, and hormones (Rayne and Aula 2020, Katuwal *et al* 2023), and air pollutants such as greenhouse gas emissions (GHGs) (e.g. methane and nitrous oxide), hydrogen sulfide, ammonia, and particulate matter (Hassouna *et al* 2016, Ward *et al* 2024). All of these pollutants can be highly concentrated as facilities tend to cluster in space for economic reasons (Miralha *et al* 2022), which can lead to differential impacts on human health. Climate change may exacerbate some of these specific issues and may influence future locations of livestock production (Rust 2019). Importantly, a growing body of evidence shows that in some regions, exposure and associated health impacts are experienced by some communities more than others, primarily racial/ethnic minority and low-income persons, raising environmental injustice concerns (Son *et al* 2024). Pollutants are not the only concern; as confined livestock operations have increased, so has the potential for zoonotic disease transfer. The recent spread of the avian flu H5N1 virus to a human exposed to dairy cattle infected with the virus (Al-Tawfiq *et al* 2024) is just one example of the many ways that livestock can facilitate the transfer of disease. Meat processing facilities even played a role in the spread of COVID-19 in the early stages of the pandemic (Taylor *et al* 2020). Further, understanding livestock operations is also critical to land-use, animal welfare, supply chain and related infrastructure, and economic planning (Weersink *et al* 2021).

Given livestock impacts and issues have been well-documented in the scientific literature, it is surprising how little data and information are available on the location, numbers, and management of livestock and their waste products (Flynn *et al* 2023). Therefore, the motivation of this perspective is to first briefly describe the limitations of existing U.S. livestock data, then to feature innovative solutions to fill data gaps, and finally to highlight the urgent need for detailed livestock data for improved public health and environmental management.

## 2. Current availability of livestock data in the U.S

In the U.S., the most comprehensive dataset for livestock is the quinquennial National Agriculture Statistics Service’s (NASS) Census of agriculture. This agricultural Census dates back to 1840, with the most recent Census occurring in 2022^17^17USDA National Agricultural Statistics Service 2022 Census of Agriculture Online: www.nass.usda.gov/AgCensus.. All data provided in the Census are self-reported by farmers through a comprehensive and well-tested survey instrument and data are quite detailed. For example, tables 12–33 of the most recent Census include inventories and sales of livestock and inventories of livestock farms for major livestock categories (cattle and calves, beef cows, milk cows, hogs and pigs, sheep and lambs, goats, kids, and mohairs, equine, poultry, and aquaculture). However, to protect farmer identity, these numbers are aggregated to a county level and, if publishing a number may reveal the identity of an operation, the data are censored for anonymity. Farmer participation in the Census has declined in recent years^18^18www.porkbusiness.com/news/industry/reasons-fewer-farmers-are-now-responding-usdas-nass-surveys-and-impact-waning., and recently NASS announced they would discontinue some of its surveys and reports^19^19www.nass.usda.gov/Newsroom/Notices/2024/04-09-2024.php., raising concerns about these data’s longevity. While data provided by the Census are inherently useful and have been used extensively by researchers, they cannot capture the range of complexities within current livestock systems and may introduce bias because of unequal reporting. For example, a broiler poultry operation may turnover their flock as often as every six weeks. While some of this may be captured among sales data, challenges arise such as accounting for a large stock requiring culling due to a disease outbreak. Numerous transfers also occur within the livestock system itself; for example, many farms grow feeder stock to be sold to finishing farms. As a result of these fine-scale changes, there are many instances where a snapshot recorded once every five years cannot account for the intricacies within livestock systems in the U.S. This is further complicated if the dataset is incomplete and thus not representative of the region.

Federal and state regulations and permits sometimes provide opportunities to access data with higher spatiotemporal resolutions. For example, in many states, livestock facilities that meet the federal definition of a CAFO may be required to obtain a National Pollutant Discharge Elimination System permit. In these cases, the permit information may be publicly available, or obtainable through a public records request, and can provide a geospatial location of the facility and annual livestock inventories. However, the complexities related to federal regulations have led to a patchwork of state-level interpretations (Rosov *et al* 2020), resulting in inconsistencies or a lack of certain data among the states. Even when the data are available, they are often in photo-copied PDFs of handwritten forms, which make data entry, extraction, and automated use difficult. Further, data include the number of animals for which the facility is permitted, not the actual number present or how that value changes across time. Regulations and data collection focused on large, concentrated feeding facilities will not capture smaller operations or grazing systems, both of which can still have adverse impacts on the environment and human health. For example, a recent study suggests that clusters of small animal operations could have a comparable impact on water quality to large operations (Miralha *et al* 2022). Additionally, there is risk of zoonotic disease even within operations that are smaller in scale (Ayala *et al* 2020).

The data described above are focused on livestock *counts*. However, equally important to understanding the impact of livestock operations is the type of animal and waste management implemented within livestock facilities (Flynn *et al* 2023). There are many ways to rear livestock in confinement that affect waste emissions including variations in feed choices, supplements and medicines provided, waste storage and use, barn cleaning and maintenance, etc. Variations in operation management have also changed over time such that facilities permitted more recently may have different design standards than older operations. Some of this information may be found in permitting documents, but again, these data face the same access and extraction issues as location and inventory data. Finally, the permitting data often provide a location that is typically the mailing address of the operation and not indicative of the exact location of livestock (e.g. barn or feedlot), spatial extent of the facility, or locations of waste storage infrastructure, which is important in exposure studies.

## 3. Emerging approaches to improve livestock data

With a clear need for better resolution of spatiotemporal livestock data, researchers and practitioners have developed novel methods to derive information to supplement the use of farmer-reported survey data. We describe a few of these approaches in the section below.

### 3.1. Identifying livestock facilities

**Figure 1.**
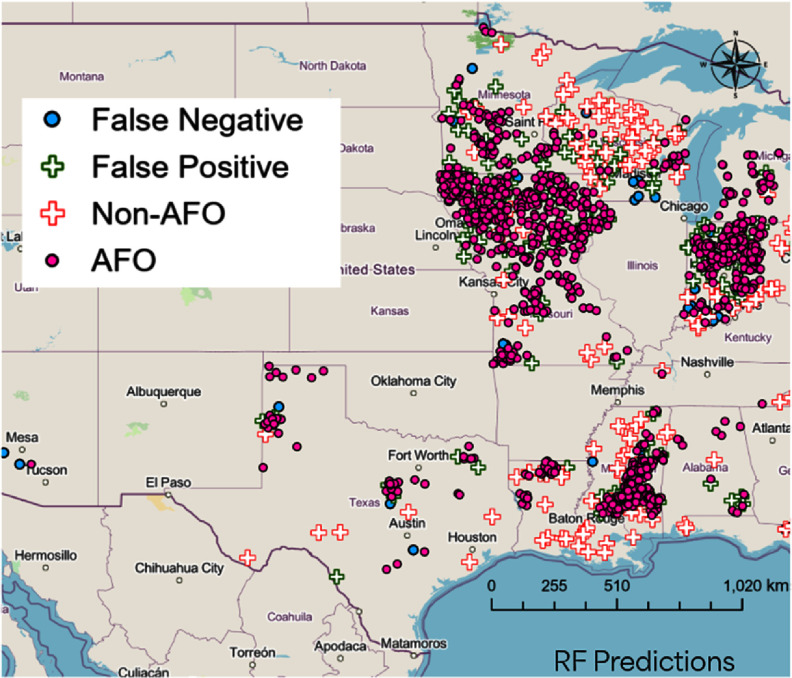
Outcomes from a random forest (RF) model using only environmental and land cover data to predict animal feeding operation (AFO) locations. The model results showed an accuracy of 87% in predicting the AFO facilities, with a precision of 89%. More details are provided in the SI.

Much of the scientific literature on livestock data curation has focused on applying remote sensing methods to identify livestock operations. Manual approaches employed by some nongovernmental organizations involve user intervention via aerial image interpretation to identify active facilities^20^20www.ewg.org/research/exposing-fields-filth., while more automated approaches may include the use of machine or deep learning to iterate through terabytes of data to identify facilities (Handan-Nader and Ho 2019, Robinson *et al* 2021). Manual approaches are time-consuming and challenges arise among automated methods as they often require extensive imagery data that are subject to their own shortcomings (e.g. cloud cover) and may have difficulty in identifying operations given variability by livestock type and state (e.g. dairy operations are structurally different than swine facilities). There are also challenges in datasets derived without consent or participation from farmers (e.g. from remote sensing-based methods) that could hinder cooperation on future mitigation options. Recent approaches combining environmental and land use data show some promise for identifying livestock facilities without aerial images (figure 1), but they also show large variability across geographic regions, highlighting the need for a finer spatiotemporal resolution in reported data. Methodologies developed in wildlife conservation spaces could potentially also be used to monitor animal counts (Tuia *et al* 2022).

### 3.2. Going beyond livestock facility location

**Figure 2.**
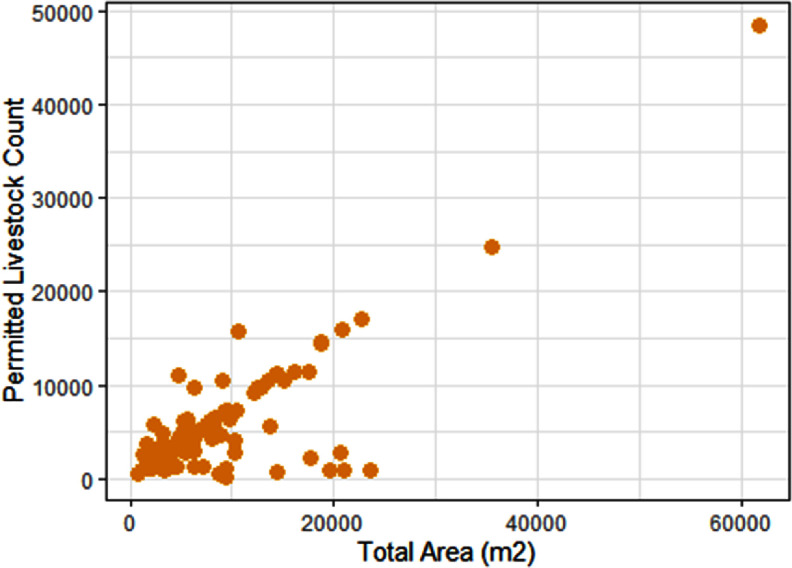
Comparison of livestock facility structure’s area and permitted animal counts for swine feeder to finish operations in Duplin County, NC. More details are provided in the SI.

**Figure 3.**
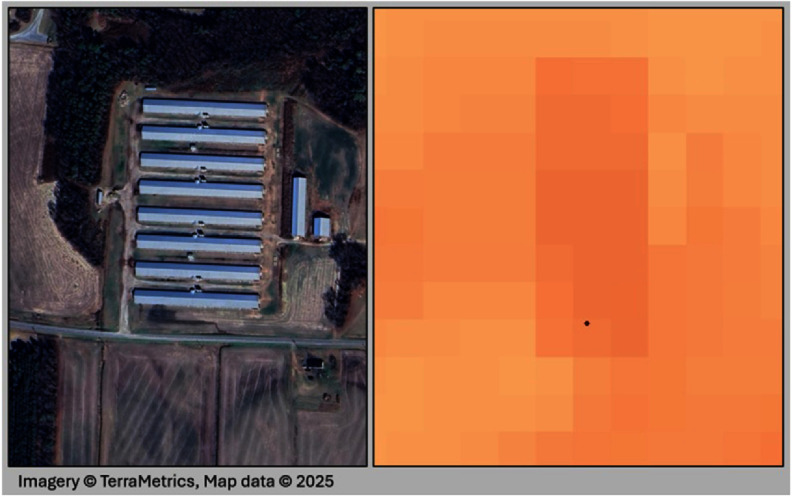
Google Earth image of an active barn in North Carolina (left) and associated thermal signature (right) from Landsat thermal bands. More details are provided in the SI.

Simply knowing locations of facilities is not enough to facilitate the comprehensive evaluation of livestock operations’ effect on the environment and human health. Identified barns could be vacant, or operating under or over their identified capacity, thereby impacting their waste emissions and manure handling. Therefore, in addition to barn locations, the number and type of animals in barns, and where, when, and how waste is being stored, treated, and applied (if at all) would improve our ability to assess health and environmental risks. Some progress is being made on this front. In recent work, soil moisture data along with other satellite-derived co-variates were used to identify swine manure applications and frequencies in North Carolina (Shea *et al* 2022). Preliminary work also suggests that the size of livestock structures can be used to estimate permitted animal counts (figure 2), though this may vary by animal type. This approach could be utilized to obtain better estimates of barn capacity and subsequent waste emissions at identified facility locations at a large geographic scale. There has also been work to examine the utility of satellite-based thermal bands as an indicator of animal presence within barns (figure 3). It should be noted that these techniques have been applied to smaller geographies with high-quality validation data derived from regulatory permits, but there is promise that these techniques could help address key data gaps. However, there is still a dire need to advance the underlying, publicly-available livestock data to help in these efforts and advance research and societal needs.

## 4. Future outlook and needs for livestock data

Despite the innovation occurring towards improving livestock data, there is a strong need to enhance livestock data collection in the U.S. and beyond to improve environmental and public health outcomes. This lack of data substantially hinders our ability to estimate public health outcomes from occupational or community exposure to livestock facilities, accurately estimate potential environmental health disparities for disadvantaged subpopulations, track and mitigate GHG emissions, and develop comprehensive nutrient management solutions like the development of a circular bioeconomy (Wainaina *et al* 2020). To truly advance our understanding of these impacts and develop mitigating solutions we call for:

1. More cohesive and centralized repositories of livestock data, including comprehensive and aligned metadata.
2. Livestock data that go beyond animal inventories and includes data on overall system management (e.g. waste handling, animal feed, pharmaceutical inputs).
3. Livestock data that are reported frequently, aligning with sub-yearly changes to animal populations.
4. Livestock data that are spatially resolved, including more details on the location of various components of operations, inter-operation livestock transfers, and manure transfers.
5. Prioritization of funding and research to enhance reported information and fill these key data gaps.

While this perspective is focused on the U.S., many of these sentiments could apply to global livestock production and thus warrant further consideration. The United Nations Framework Convention on Climate Change, for example, also requires GHG emissions reporting under Annex 1 that includes the data discussed here (Ward *et al* 2024), demonstrating an international need. Addressing the identified gaps in livestock data in the U.S. will likely require changes to policies and regulations that may be a difficult and slow process, but a necessary one to address extant and future issues caused by the growing livestock industry on environment, climate, and health. We suggest the integration and collaboration of livestock producers and other interested and affected parties to enable a process that can address concerns over privacy while simultaneously allowing for the comprehensive evaluation and alignment of livestock data with environmental and public health concerns. Incorporating a level of trust and transparency for the livestock system can help to provide the livestock products that are relied upon while simultaneously protecting environmental and human health.

## Data Availability

All data that support the findings of this study are included within the article (and any supplementary files).
